# The current state of demographic subgroup reporting for commercially available AI for radiology: a scoping review

**DOI:** 10.1007/s00330-026-12652-y

**Published:** 2026-06-12

**Authors:** Shannon L. Walston, Hirotaka Takita, Yasuhito Mitsuyama, Junya Sato, Daiju Ueda

**Affiliations:** 1 Department of Artificial Intelligence, Graduate School of Medicine, Osaka Metropolitan University, Osaka, Japan; 2 Department of Diagnostic and Interventional Radiology, Graduate School of Medicine, Osaka Metropolitan University, Osaka, Japan; 3 Department of Artificial Intelligence in Diagnostic Radiology, The University of Osaka, Graduate School of Medicine, Suita, Japan

**Keywords:** Artificial intelligence, Radiology, Radiographic image interpretation, Computer-assisted, Odds ratio, Secondary data analysis

## Abstract

**Objective:**

Though subgroup performance reporting helps ensure the safety of artificial intelligence (AI) products, the extent of this reporting remains unclear. This scoping review identifies studies validating commercially available AI-based products and reports the trends in performance reporting across sex, age, and race/ethnicity demographic subgroups.

**Materials and methods:**

Peer-reviewed validation studies of commercially available products published after 2010 were collected from the Health AI Register and PubMed on 29 November 2024. Study trends in the reporting of sex, age, and race/ethnicity were mapped with regression analysis. We apply the Wilson confidence interval equation to estimate which tuberculosis detection studies are underpowered for subgroup meta-analysis.

**Results:**

Three hundred ninety-two of 545 studies validating 252 products reported subgroup demographic data for any of the three groups. Only 77 of these presented subgroup performance results. Skeletal (20/88) and lung (30/139) studies, and those utilizing chest (24/79) or bone (19/63) radiographs, most often presented subgroup performance data. We found no evidence that more recent studies (OR: 1.039 [95% CI: 0.959–1.127]) or company sponsorship (OR: 1.010 [95% CI: 0.492–1.920]) led to increased subgroup reporting. We show that 14/21 tuberculosis datasets may be underpowered for post-hoc subgroup meta-analysis.

**Conclusion:**

This scoping review quantifies how fragmented the commercial validation landscape is, showing that reporting for both the demographics and per-subgroup performance is inadequate for estimating subgroup bias. This systemic problem requires effort from all stakeholders, from researchers to regulatory agencies, encouraging thorough reporting and commercial product validation to support physician and patient trust in medical AI products.

**Key Points:**

***Question*** *The number of studies validating the performance of each commercially available radiology AI product for minority subgroup bias is unclear*.

***Findings*** *The currently available commercial AI validation studies often neglect to describe demographic subgroup data, and fewer provide performance results per subgroup, prohibiting algorithmic bias meta-analysis*.

***Clinical relevance*** *Physician and patient trust in the medical AI already used clinically must be built on peer-reviewed literature and meta-analysis. The current literature is insufficient for determining the safety and performance of these products for demographic minorities*.

**Graphical Abstract:**

**Figure:**
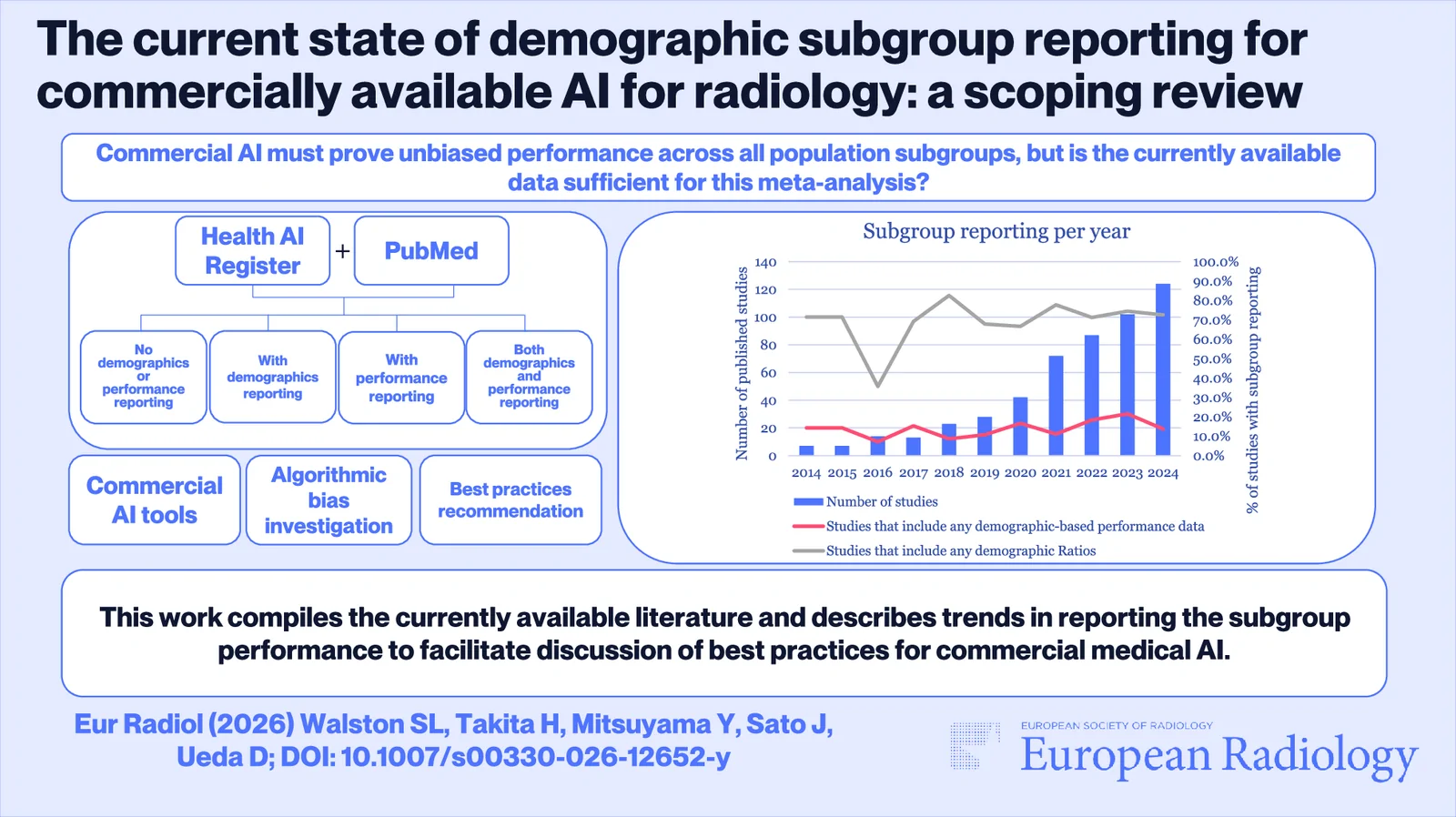

## Introduction

Artificial intelligence (AI) is a part of the growing industry of medical software products, many of which are specific to radiology. Recent commentaries and reviews have noted the risks that algorithmic bias in medical AI poses to patient subgroups distinguished by demographics, socioeconomic factors, or geographic factors [1–6]. These biases might be amplified when the model is applied to an external cohort, one that differs demographically from the highly curated testing cohorts [7]. While external validation can identify broad performance differences, these studies still lack the resolution to expose performance discrepancies between patient demographics. To date, little evidence exists proving or disproving the presence of subgroup bias in commercial medical AI [8] or showing that patient-facing models are operating within reasonable limits for even the most common patient demographic subgroups, such as sex, age, and race/ethnicity.

Even though we know AI can identify patient demographic subgroups with or without training [9], and even though we know the algorithm may use these subgroups as a shortcut [10] instead of performing the intended task, few rules or regulations exist to address this. Approval from regulatory organizations such as the US Food and Drug Administration (FDA) or the presence of the CE mark under the EU MDR indicates that the product has been shown to perform appropriately for its intended use, not that it is safe and unbiased for specific population subgroups. Furthermore, current regulations do not require that the evidence submitted for approval of commercial AI be also peer reviewed and published [11–13]. As medical AI models become progressively deep and complex, algorithmic biases might persist that aren’t apparent from the basic performance testing provided to regulatory organizations [14].

To date, best practices for estimating algorithmic bias in medical AI products used clinically remain unclear due to the limited information available [15, 16]. Regulatory and patient advocacy groups are preparing solutions for identifying and quantifying algorithmic bias [17, 18]. Unfortunately, such groups also struggle with the general lack of data regarding commercial medical AI [8]. This cycle, where there are no regulations because there is no data to base them on, and the data is limited and incomplete because there are no baseline regulations, leaves clinicians wary that such products harbor potentially harmful biases.

### Motivation and aims

We aimed to conduct a scoping review to (a) identify studies validating commercially available AI-based products, (b) assess how often age, sex, and race/ethnicity demographics are reported and if subgroup performance per demographic is reported, and (c) provide practical recommendations to improve such reporting to enable future meta-analysis of algorithmic bias.

## Materials and methods

### Ethical approval

This review utilized only publicly available information and, therefore, no regulatory or institutional review board approval was necessary.

### Study design

We followed the PRISMA-ScR guidelines with the updated methodological guidance for scoping reviews from the JBI [19, 20]. The protocol was developed a priori and is available in the supplement, but was not prospectively registered as PROSPERO does not collect scoping reviews.

### Data collection

Publications describing the development or validation of commercially available products were identified from an independently assembled database of CE-marked AI products called the Health AI Register and PubMed. The PubMed database was searched for the terms ‘artificial intelligence’, ‘radiology’, ‘validation’, ‘commercially available’ and their variants, as well as known company names. Commercial availability was established by identifying the product within the FDA database of AI and Machine Learning (AI/ML)-Enabled Medical Devices, or the EUDAMED database. The databases were searched on November 29, 2024, by one author, and available product information published after January 1, 2010, was collected. All relevant information for each model was input into a predesigned spreadsheet by one author. Searching details are available in the Electronic Supplementary Material.

### Inclusion/exclusion criteria

Studies were included if they evaluated the performance of a commercially available product that incorporated an AI element, as defined by the International Organization for Standardization [21], that AI element utilized radiological imaging, and the product was externally tested in a human patient cohort. Performance was defined as a numerical evaluation comparing the product output to that of a standard, including sensitivity, error, AUC, or similar values. AI elements are those with trainable parameters that can be updated or fine-tuned, where non-AI machine learning elements are all other algorithms, such as watershed image processing, deformable image registration, and calibration curve alignment. Studies published prior to product naming and marketing were included if the product could be determined and they fulfilled the inclusion criteria, as these studies are often included on marketing materials and databases such as the Health AI Register.

Studies were excluded that tested a hypothesis unrelated to validating product performance, such as utilizing the model output as a quantitative standard or benchmark for novel tool development [22, 23], because these assume appropriate performance of the product. Cost effectiveness [24] and workflow optimization [25, 26] studies were also excluded. Whitepapers, briefings, or other similar documents, abstracts, case reports, review articles, videos, and presentations, broken links, retracted papers, and posters were excluded.

### Data extraction

Data was collected into a prepared Google Sheets workbook shared with all authors. The primary outcomes were the reporting of patient demographic ratios of the external testing set and the reporting of model performance metrics for available demographic groups. The patient demographics targeted in this study were those previously identified as the most commonly reported: sex, age, and race/ethnicity [27]. As in previous reviews [5], data for each product were recorded in the product-level spreadsheet for studies that provided results for multiple products. The study-level spreadsheet was used for publication evaluations, and the product-level spreadsheet was used for product-level evaluations. As each product is only represented once per study, even when there are multiple products, there is no risk of multiple observations per study or non-independence in product-level evaluations. The study was classified as presenting patient subgroup demographics if the number of patients was split based on sex, age, or race/ethnicity into at least two groups. The study was classified as presenting subgroup results if there were numerical values recorded for the predefined, relevant metric for two or more demographic-based (sex, age, or race/ethnicity) groupings of patients. Study design information, such as the publication year, organ system evaluated, the imaging modalities tested, author COIs, and study sponsorship, was included to assess sources of heterogeneity. Sponsorship of the study by any of the manufacturers of the products being validated was recorded from the article text. Author associations with the manufacturers of the products being validated were determined based on their affiliations and conflicts of interest statements in the text or if they were listed as employees or staff on the manufacturer's website. These relationships included employment, ownership, stock options, or payments.

### Data synthesis

Findings per study and per product were first summarized using descriptive statistics. Binomial logistic regression models were fitted per study, with publication year held as the continuous predictor variable and inclusion of demographic data or results per demographic held as the dependent variable. Multivariable binomial logistic regression was used per study to determine if study sponsorship or author associations predicted whether demographics or results per demographic would be reported while controlling for temporal trends via publication year. For binomial performance measures such as accuracy or sensitivity, the minimum performance (Smin) per subgroup was calculated by dividing the complement of the overall product performance measure (Soverall) by the prevalence of the subgroup in the test dataset and subtracting this from 1. (Smin = 1 − [(1 − Soverall)/*x*])

The number of patients per subgroup needed to attain a certain metric can be roughly estimated using the Wilson confidence interval (CI) equation [28–30]. To demonstrate this technique, we determined post-hoc that the studies that validated products for TB detection most often included the disease prevalence necessary for this calculation. Adjusting for the slight imbalance between the number of patients per sex didn’t significantly affect the results, so we did not adjust the equation.

Details of these equations can be found in the Electronic Supplementary Material. Significance for statistical analysis was set at 0.05. All analyses were performed using R (version 4.2.2).

## Results

### Included studies

**Fig. 1:**
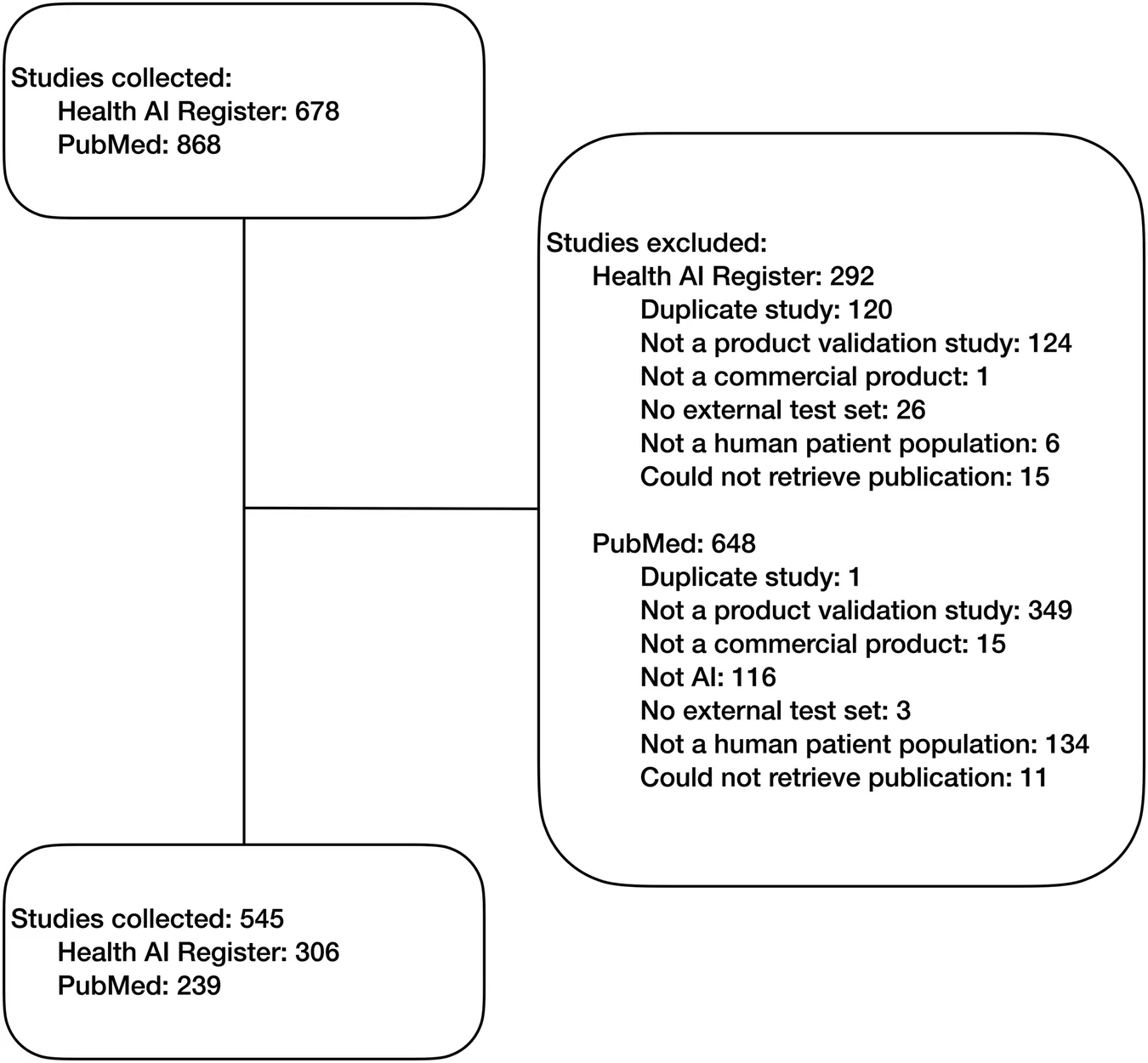
Study collection and inclusion flowchart

**Fig. 2:**
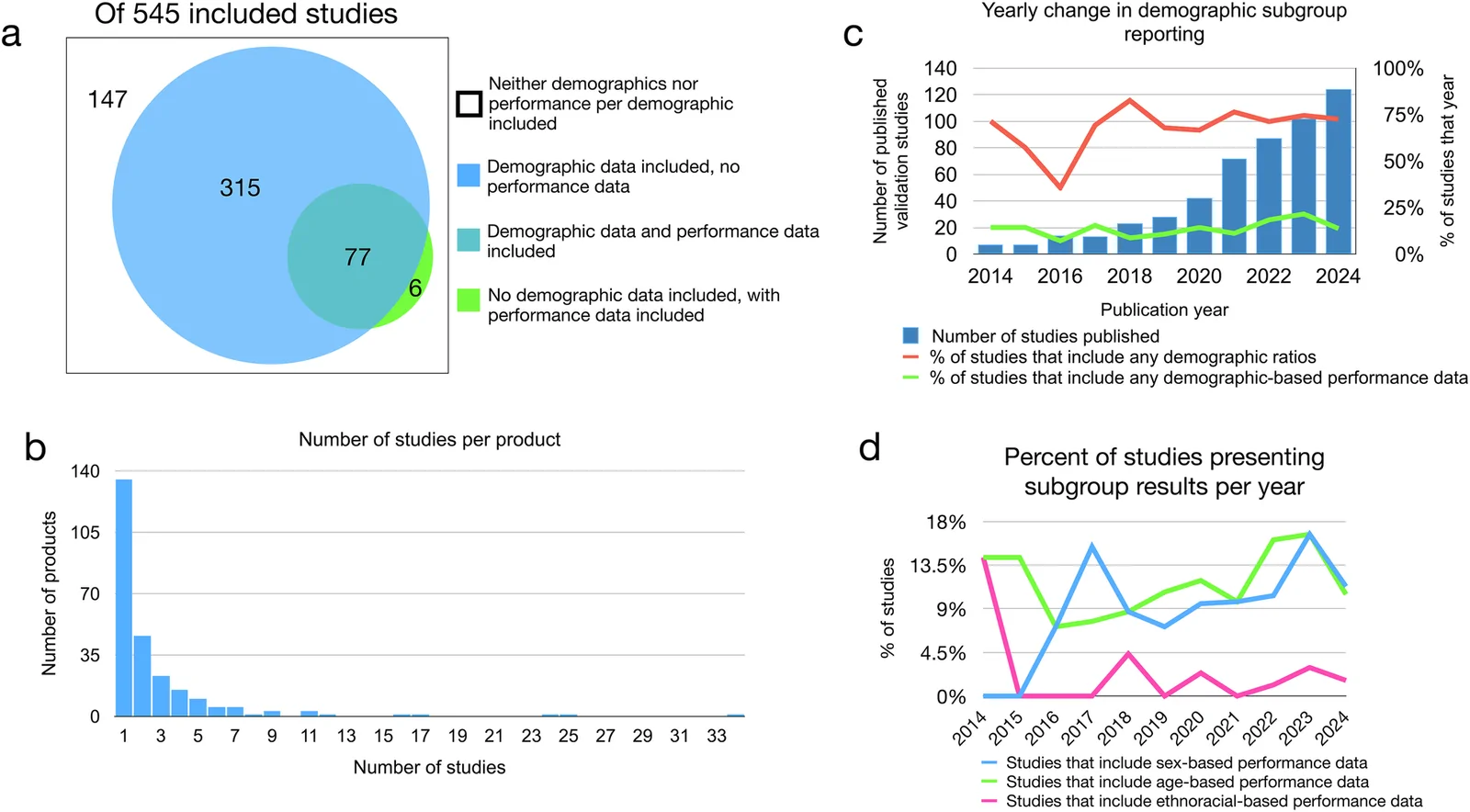
Demographic subgroup reporting overview. **a** Venn diagram of the included studies. The blue circle represents studies that included the number of patients per either age, sex, or race/ethnicity. (*n* = 392) The green circle represents studies that included the performance of the AI product for each group for either age, sex, or race/ethnicity. (*n* = 83) The teal circle represents where these overlap; studies that included both the number of patients and the performance of the AI product for either age, sex, or race/ethnicity. (*n* = 77). **b** Publication frequency by product. The *x*-axis represents the number of validation studies, and the *y*-axis represents the number of products with that number of validation studies. Most products (*n* = 135) had only one validation study. There were 9 products that were validated in ten or more studies. **c** Temporal changes in demographic subgroup reporting. The percent of studies per publication year that report the number of patients (orange) or the product performance (green) in any subgroup (sex, age, or race/ethnicity). The blue bars represent the number of validation studies published that year. **d** The percent of studies per publication year that report the product performance per subgroup (sex (blue), age (green), or race/ethnicity (pink))

There were 868 records collected from the PubMed database and 678 records pulled from the Health AI Register. The search resulted in 545 eligible validation studies: 306 eligible studies from the Health AI Register and 239 eligible studies from PubMed (Table 1). The inclusion flowchart is shown in Fig. 1. Subgroup analysis results were presented in 83 studies, but six of these studies did not include the patient count for the evaluated subgroups. Thus, 77 of 545 validation studies presented both demographic details and subgroup analysis results for that demographic subgroup (Fig. 2a). These 77 studies validate the performance of 52 commercially available products from 38 manufacturers (Fig. 2b). There were between one and 34 studies per product, with nine products having 10 or more studies. The most evaluated product, Lunit Insight CXR, had 16/34 studies that included the performance of any subgroup.

**Table 1:** Summary of the included studies

	# Studies	All demographic groups		Sex		Age		Race/ethnicity	
		Studies that include any demographic Ratios	Studies that include any demographic-based performance data	Ratios	Performance Results	Ratios	Performance Results	Ratios	Performance Results
Health AI Register	306 (56.1%)	217 (70.9%) (95% CI: 0.6548–0.7594)	48 (15.7%) (95% CI: 0.1180–0.1925)	201 (65.7%) (95% CI: 0.6007–0.7099)	35 (11.4%) (95% CI: 0.0810–0.1555)	48 (15.7%) (95% CI: 0.1180–0.2025)	41 (13.4%) (95% CI: 0.0979–0.1773)	27 (8.8%) (95% CI: 0.0590–0.1258)	7 (2.3%) (95% CI: 0.0092–0.0466)
PubMed	239 (43.9%)	175 (73.2%) (95% CI: 0.6713–0.7873)	35 (14.6%) (95% CI: 0.1042–0.1977)	165 (69.0%) (95% CI: 0.6276–0.7484)	27 (11.3%) (95% CI: 0.0758–0.1601)	31 (13.0%) (95% CI: 0.0899–0.1790)	26 (10.9%) (95% CI: 0.0723–0.1553)	18 (7.5%) (95% CI: 0.0452–0.1164)	2 (0.8%) (95% CI: 0.0010–0.0299)
TOTAL	545	392	83^*^	366	62	79	67	45	9

^*^Six studies do not include the number of patients in the evaluated demographic group

#### Temporal trends

Though there are a larger number of studies which include subgroup evaluation in more recent years, more recent studies are not significantly more likely to include subgroup analysis (OR: 1.039 (95% CI: 0.959– 1.127), *p* = 0.347). (Fig. 2c) This is also true for all subgroups (Table 2 and Fig. 2d).

**Table 2:** Odds ratios (ORs) for the reporting of subgroup demographics and performance metrics per year

Odds of more recent studies	OR per publication year	Estimate	Std. error	*z* value	Pr (>|*z*|)
Reporting any demographics	1.065	0.06275	0.02893	2.169	0.030
Reporting sex demographics	1.055	0.05	0.03573	1.505	0.132
Reporting age demographics	1.132	0.12	0.0504	2.466	0.0137
Reporting race/ethnicity demographics	0.939	−0.06	0.04652	−1.361	0.174
Reporting performance results	1.039	0.03867	0.04115	0.94	0.347
Reporting sex analysis	1.034	0.03356	0.04744	0.708	0.479
Reporting age analysis	1.059	0.05712	0.04715	1.211	0.226
Reporting race/ethnicity analysis	0.916	−0.08733	0.08271	−1.056	0.291

#### Reporting on demographic characteristics and performance

The sex of the patients in the test set was recorded for 366 (81%) of 453 studies. This value excludes 92 sex-specific studies, such as those of the breast or prostate, because sex-based performance comparison is not applicable. Of these, 59/366 (16%) provided sex subgroup performance data.

There were 79/545 (14%) studies that recorded patient age information divided between two or more age groups. Though 67/545 studies provided product performance measures across stratified age groups, 27 of these did not include the number of patients per age group. Only 40/79 studies (51%) provided the number of patients in each of the age groups for which they reported performance measures.

Race/ethnicity ratios were reported for 45 (8%) studies; 12 of which acknowledged their cohort primarily comprised one race/ethnicity group. There were 8/45 that reported both the number of patients and the model performance per race/ethnicity group.

Performance reporting for any demographic subgroup did not exceed 30% of studies for any radiology subspecialty (0%–23%) or imaging modality (0%–30%) (Table 3).

**Table 3:** Summary of the included studies per subspecialty/organ system and imaging modality

	# Studies	With subgroup details reporting	%	With performance reporting	%	% that this comprises of 545 studies
Subspecialty/organ						
Skeletal	88	73	83.0%	20	22.7%	16%
Lung	139	116	83.5%	30	21.6%	26%
Abdomen	11	9	81.8%	2	18.2%	2%
Breast	73	24	32.9%	11	15.1%	13%
Cardiology	68	60	88.2%	7	10.3%	12%
Brain	117	86	73.5%	10	8.5%	21%
Liver	15	12	80.0%	1	6.7%	3%
Genitourinary	6	2	33.3%	0	0.0%	1%
Lymph	2	2	100.0%	0	0.0%	0%
Prostate	16	0	0.0%	0	0.0%	3%
Thyroid	5	5	100.0%	0	0.0%	1%
Various	5	3	60.0%	2	40.0%	1%
Imaging modality						
CXR	79	66	83.5%	24	30%	14%
Radiography	63	53	84.1%	19	30%	12%
Mammography	62	21	33.9%	10	16%	11%
CT	215	170	79.1%	21	10%	39%
MRI	90	62	68.9%	7	8%	17%
Ultrasound	27	15	55.6%	2	7%	5%
PET	5	3	60.0%	0	0%	1%
CT or MRI	2	1	50.0%	0	0%	0%
Scintigraphy	2	1	50.0%	0	0%	0%

#### Influence of funding or conflicts of interest on subgroup analysis

Funding details were described in 458/545 studies, and these were utilized to evaluate if sponsorship might be related to more rigorous reporting and subgroup evaluation. The study was sponsored by the manufacturer of the product being validated in 78/458 (17%) studies. We did not find a significant relationship between subgroup results reporting and funding provided by the product manufacturer (OR: 1.010 (95% CI: 0.492–1.92)).

The authors had a direct relationship with the manufacturer of the product being investigated in 269 studies and no evident relationship in the remaining 276 studies. We did not find a significant relationship between subgroup results reporting and direct relationships with the product manufacturer (OR: 0.736 (95% CI: 0.437–1.23)).

#### Estimation of the minimum possible subgroup performance

Mathematically, the potential range of performance measures is inversely related to the ratio of the subgroup to the whole cohort. For example, we collected 64 sensitivity values for the Lunit Insight CXR product across 27 studies. The collected studies averaged 84% sensitivity with a median of 84% sensitivity. If we apply the minimum performance formula to the sex demographic, where female patients averaged 47% of the cohort, we get the formula Smin = 1 − ((1 − 0.84))/0.47. Solving for Smin, we get 65.96%. Thus, the sensitivity for the female subgroup can be as low as 66% while maintaining an overall sensitivity of 84%. Details of these calculations are in the Electronic Supplementary Material.

This equation can also be used for subgroups with multiple categories, such as race/ethnicity. For example, from our data, a typical dataset comprises 64.8% White patients, but the next largest ethnic group, Asian patients, comprises a normalized median of 14% of all recorded datasets. Substituting 0.14 as the denominator in (Smin = 1 − [(1 − Soverall)/^*x*^]) dramatically widens the potential performance range for the Asian subgroup to 0% for overall sensitivities below 85%. This issue is further exaggerated with less represented subgroups.

#### Patients required per subgroup for subgroup analysis

The number of patients per subgroup needed to attain a certain sensitivity was roughly estimated using the Wilson CI equation detailed in the Electronic Supplementary Material. For unbalanced groups like race/ethnicity demographics, include the variable k: the number of patients in subgroup 2 divided by the number of patients in subgroup 1, as detailed in the Electronic Supplementary Material.

Following the work of Bosman et al [31], their population had a 10% TB prevalence, and they targeted a sensitivity near 90% as recommended by the WHO [32]. If we assume reasonable values such as a precision of 5%, power at 80%, and a half-width of 5%, the Wilson CI formula gives us that each dataset must have at least 139 TB positive patients in both sex subgroups. Meaning there must be a total of 1399 patients per sex at this prevalence, or at least 2798 total patients to attain this minimum. Thus, we suspect this study is underpowered for sex subgroup analysis with only 1232 patients total, 139 of which were TB-positive patients (Table 4).

**Table 4:** Number of patients per sex for studies validating commercial AI for TB detection

DOI	No. of patients	TB prevalence (TB positive/total patients)	# Male	# Female	Reported overall sensitivity	Threshold at TB prevalence and reported sensitivity (patients per sex required)	Threshold at TB prevalence and 90% sensitivity (patients per sex required)
https://doi.org/10.1093/cid/ciy967	132	39.4%	57	79	94%	211	355
https://doi.org/10.1093/cid/ciy967	140	50.0%	66	74	94%	166	280
https://doi.org/10.1093/cid/ciy967	170	41.2%	92	88	94%	202	340
https://doi.org/10.1093/cid/ciy967	173	59.5%	92	81	94%	140	235
https://doi.org/10.1093/cid/ciy967	183	45.4%	101	82	94%	183	309
https://doi.org/10.1016/S2589-7500(20)30221-1	272	12.4%	130	142	93%	815	1131
https://doi.org/10.1016/S2589-7500(24)00118-3	774	33.3%	396	378	90%	408	420
https://doi.org/10.1038/s41598-021-03265-0	1032	12.9%	712	320	96%	579	1086
https://doi.org/10.1038/s41598-019-51503-3	1196	9.1%	550	646	91%	1396	1535
https://doi.org/10.1093/cid/ciae378	1232	10.0%	553	679	90%	1399	1399
https://doi.org/10.1016/j.ijid.2022.05.037	2190	12.3%	1144	1046	88%	1338	1139
https://doi.org/10.1007/s00330-022-09315-z	3047	0.2%	2162	885	65%	217,237	85,278
https://doi.org/10.3348/kjr.2022.0189	5887	0.3%	4329	1558	97%	14,716	51,489
https://doi.org/10.1093/cid/ciy967	646	49.5%	440	206	94%	168	283
https://doi.org/10.1038/s41598-019-56589-3	929	51.8%	647	282	71%	621	270
https://doi.org/10.1371/journal.pgph.0000402	2812	30.1%	1555	1257	84%	689	465
https://doi.org/10.1093/cid/ciab639	3727	17.0%	1769	1583	90%	823	823
https://doi.org/10.1016/j.ijid.2024.107221	4733	17.1%	2626	2107	90%	818	818
https://doi.org/10.1038/s41598-020-62148-y	5565	15.3%	2756	2809	90%	915	915
https://doi.org/10.1371/journal.pdig.0000067	12,890	19.0%	8765	4125	88%	840	737
https://doi.org/10.1016/S2589-7500(21)00116-3	23,954	15.3%	16,078	7876	92%	789	915

Continuing with the tuberculosis detection example, there were 16 total studies that described 21 datasets with variable TB prevalence ranges. It is unclear if this is near the true population prevalence, but the dataset prevalence is adequate for the sake of this example (Table 4).

From the table, we see that 14 (67%) of the 21 datasets are likely underpowered for sex subgroup meta-analysis. Per product, there are not enough studies to allow adequately powered subgroup meta-analysis. We found ten studies evaluating the Delft Imaging CAD4TB product, however, only 6 of these had enough patients in both sex subgroups to achieve our generous threshold in this example.

## Discussion

Though measurement and mitigation of algorithmic bias is an important goal, we must first focus on providing the relevant data that underpin those discussions. We can’t do calculations without data, after all, and this review is not the first to note fewer studies than should be expected [33]. In this scoping review, we collected 545 studies validating 252 commercially available AI products from 142 manufacturers. Only 14% (77/545) of these included the performance of the AI for any of the three most common demographic subgroups: sex, age, or race/ethnicity [27]. Importantly, we show that the performance reporting for demographic subgroups has not become more common in recent years, despite the numerous calls for improved demographics reporting to address the dangers of biased medical AI [1–5, 18].

### Bias risk demonstrations

#### Estimation of minimum subgroup performance

Most studies we collected aimed to validate overall population performance and not subgroup performance measures [34], and we are not questioning their published claims. To demonstrate that reporting only overall performance is insufficient, we applied a minimum subgroup performance estimation equation. This equation allows us to visualize how quickly the statistically possible performance range becomes too wide to be useful for minority subgroups [35]. The current lack of published and reproduced subgroup performance data casts reasonable doubt on the representativeness of the clinical product performance for underrepresented subgroups.

#### Patients required per subgroup for subgroup analysis

Though subgroup imbalance theoretically expands the range of performance measures, the number of patients in the subgroups is actually important. Datasets with enough patients per minority group can achieve adequate statistical power to provide clinically meaningful insight into the AI performance for that subgroup, even at a low overall ratio of the population.

The Wilson CI formula relates the disease prevalence, the target metric, the target statistical power, and the number of patients. This formula works with any binomial value, so we can substitute sensitivity for any appropriate binomial value, such as accuracy or specificity. Applying the Wilson CI formula to studies validating AI for tuberculosis detection, we showed that 67% of the reported datasets are at risk of being underpowered for sex subgroup analysis. TB detection was chosen for this analysis out of convenience because the target sensitivity is standardized, and the prevalence is often reported. The published performance in these studies is not in question, but these examples justify our desire for the performance of population subgroups to be reported.

### Strategies for improving AI-based product validation studies

We did not detect an association between time on the market and demographic reporting or subgroup analysis, so study sponsorship, authorship conflicts of interest, and other factors, such as the fact that authors may be unaware of the unique reporting needs for studies of AI products. To help enable future algorithmic bias evaluation, we have identified four points that can be addressed immediately, such as the choice of demographic variables to report, funding struggles and time constraints, the limited data availability, and the use of specialized keywords to improve visibility. We offer these recommendations to support stakeholders across the medical AI landscape in improving the definitions and reporting of data necessary and relevant for algorithmic bias analysis.

First, detailed demographic information not considered relevant to the hypothesis may not be included in the publication. Unfortunately, AI models do not necessarily ‘learn’ the same biases as humans, so our assumptions about what demographics are relevant may not match those that the model adopted during training. Our estimations of the minimum subgroup performance show that readers should consider that underrepresented subgroups may have lower performance than the overall metric states. Not only might the model utilize provided demographic variables, but previous work has shown that AI can distinguish demographic variables such as sex and race/ethnicity without specific training [9, 10], and models can show unexpected biases based on this data. We recommend that demographic data be provided even when variability is not expected for the clinical task [18, 36–38].

Second, financial considerations may be a factor limiting the publication of studies validating products known to be used in hundreds of facilities [5]. These may not be considered “novel,” “transformative,” or any of the terms favored by the top journals despite their high clinical relevance, reducing the fervor for performing them and often relegating such work to expensive open-access journals. Though individual facilities may not have the resources to publish research [39], advocacy groups and manufacturers do [39, 40]. A collaborative space, such as a public-private data quality assurance partnership or an external quality assurance lab network, may help facilities connect with groups interested in evaluating the performance of these products across various clinical settings [39, 40]. We recommend a shift towards encouraging the publication of studies validating commercially available models, even when funded by the manufacturer. Regardless of the funding source, future validation studies should use the most up-to-date reporting standards, such as FUTURE-AI [41], MAIC-10 [42], TRIPOD-AI [43], and the anticipated PROBAST-AI [44] to improve transparency, reporting quality, and bias assessment.

Third, patient data availability may limit reporting in validation studies. Patients have the right to privacy and may choose not to disclose protected information, so recommending more rigorous data collection is as impractical as it is unhelpful. Instead, authors can support downstream bias analysis by performing a secondary validation test with a benchmarking dataset or presenting known data in a standardized format and clearly labeling unknown data for each demographic.

When authors are faced with missing demographic values in their local cohort, they can support their validation study with a benchmarking analysis on a dataset sized per the Wilson CI formula. The benchmark’s subgroup-level analysis may reveal disparities that aggregate metrics would miss. Significant subgroup performance differences could indicate a training gap that should be discussed along with the overall clinical cohort metrics obtained in the validation study. This method is based on the hypothesis that differences in subgroup performance findings between studies can reflect training data gaps in commercial models [45]. Many current validation studies already perform such experiments for variables such as scanner manufacturer [46], clinical setting, etc., showing a path for applying this to demographic subgroups. Commercial models can be validated on local sets even in cases where patient privacy rules or data collection standards have led to missing demographic details in the available data.

We also support the trend towards standardizing subgroup reporting into a consistent and easy-to-read “facts label” style presentation [47]. Standards already in place for clinical trials, such as the CONSORT guidelines [48] and the NIH requirements for funded studies [49], offer a backbone for AI-based product evaluation across subspecialties. Standardizing demographic reporting for studies of regulated products would improve patient and clinician trust in commercial AI products.

Our fourth recommendation is for authors to use clear and specific manuscript keywords to facilitate discovery and meta-analysis of these commercial product validation studies. These terms should be relevant and specific, such as the name of the product or manufacturer, but general terms such as “commercial product” or “FDA approved” may also be helpful. As is apparent from our search criteria (Electronic Supplementary Material), we struggled to identify relevant studies. While MESH terms are standardized and well organized, they do not distinguish commercial and non-commercial products or those with regulatory approval [50]. Furthermore, MESH terms cannot be used to identify specific products or manufacturers.

## Limitations of this work

This review is limited in scope to the field of radiology. This scoping review does not quantify the performance difference between any products or classes of products. We did not compare these products based on any measures representing how many facilities or patients use their products. This work was designed and performed by one author, which may have led to selection bias. The required sample size estimate calculations are intended as illustrative examples to encourage further discussion and are not necessarily generalized to all clinical contexts and AI-based products.

## Conclusion

Previous reviews, editorials, and commentaries have regarded algorithmic bias as a problem requiring targeted statistical evaluation methods and better data curation, but our work shows a key roadblock is far earlier in the process: the failure to disclose critical patient demographics such as age, sex, and race/ethnicity within validation studies. We offer four recommendations to improve demographic subgroup reporting and testing in validation studies to enable future meta-analysis of commercially available products. Effective, transparent reporting is a necessary first step towards confirming the safe and unbiased performance of these medical AI products across all patient subgroups, as well as improving both physician and patient trust in medical AI products.

## Supplementary information

ELECTRONIC SUPPLEMENTARY MATERIAL

## Abbreviations

AI: Artificial intelligence
CI: Confidence interval
FDA: US Food and Drug Administration
OR: Odds ratio
