# Supplementary material for: The current state of demographic subgroup reporting for commercially available AI for radiology: a scoping review
Source: Eur Radiol. 2026 Jun 12;36(10):7512–23. doi: 10.1007/s00330-026-12652-y (PMC13574847; doi:10.1007/s00330-026-12652-y)
Supplement: Supplementary file 1 [file 330_2026_12652_MOESM1_ESM.pdf]

ELECTRONIC SUPPLEMENTARY MATERIAL

The current state of demographic subgroup reporting for commercially available AI-based products for radiology: a scoping review

Supplementary methods

a) Pre-determined data headers

b) PubMed search criteria

c) Code used to extract publications from Health AI Register database

d) Minimum sensitivity equation

e) Wilson CI equation

Supplementary tables

Table S1: Studies that present both demographic details and subgroup analysis results for that demographic subgroup

Supplementary references

a) Predesigned spreadsheet has the following columns:

Paper code
Source
DOI
Evaluates commercial product?
AI?
Radiology?
External test set?
Human test cohort?
Company name in the paper
Current company name
Product
CE-mark
FDA approved
publication year
Subspecialty / organ
Imaging Modality
Imaging Modality for tables
Study sponsored by device maker?
No. of Patients
Demographic details reported for sex, age or race
Documents include Sex Ratios
Documents include Age Ratios
Documents include Race Ratios
Metric reported for sex, age or race?
Metric for males
Metric for females
Metric for younger group
Metric for older group
Metric for White / Caucasian
Metric for Black / African
Metric for Asian
Metric for Hispanic
Metric for other

b) Pubmed search criteria:

("radiology"[MeSH Terms] OR "radiology"[All Fields] OR "radiography"[MeSH Terms] OR "radiography"[tiab] OR "radiology's"[tiab] OR "radiologists"[tiab] OR "radiologist's"[tiab] OR "radiologic"[tiab] OR "radiological"[tiab] OR "radiologist"[tiab]) AND (("Algorithms"[Mesh] OR "Artificial Intelligence"[Mesh:NoExp] OR "Machine Learning"[Mesh] OR "Deep Learning"[Mesh] OR "Artificial Intelligence"[tiab] OR "Machine Learning"[tiab] OR "neural network"[tiab] OR "neural networks"[tiab] OR "deep learning"[tiab] OR "augmented intelligence"[tiab] OR AI[Title]) AND ("hospitals"[MeSH Terms] OR "Decision Support Systems, Clinical"[Mesh] OR Health[tiab] OR "health care"[tiab] OR healthcare[tiab] OR hospitals[tiab] OR hospital[tiab] OR "medical center"[tiab] OR "medical centers"[tiab] OR "Health systems"[tiab] OR "Health system"[tiab] OR "health-care"[tiab] OR medical[tiab] OR clinical[tiab]) AND (detect[tiab] OR detects[tiab] OR detected[tiab] OR detecting[tiab] OR detection[tiab] OR Assess[tiab] OR assesses[tiab] OR assessed[tiab] OR assessing[tiab] OR

assessment[tiab] OR evaluate[tiab] OR evaluates[tiab] OR evaluated[tiab] OR evaluating[tiab] OR evaluation[tiab] OR framework[tiab] OR frameworks[tiab] OR validate[tiab] OR validates[tiab] OR validated[tiab] OR validating[tiab] OR validation[tiab] OR measure[tiab] OR measures[tiab] OR measured[tiab] OR measuring[tiab] OR measurement[tiab] OR monitor[tiab] OR monitors[tiab] OR monitored[tiab] OR monitoring[tiab] OR analyze[tiab] OR analyzes[tiab] OR analyzed[tiab] OR analyzing[tiab] OR analysis[tiab] OR analyse[tiab] OR analyses[tiab] OR analysed[tiab] OR analysing[tiab] OR screen[tiab] OR screens[tiab] OR screened[tiab] OR screening[tiab] OR report[tiab] OR reports[tiab] OR reported[tiab] OR reporting[tiab] OR compare[tiab] OR compares[tiab] OR compared[tiab] OR comparing[tiab] OR comparison[tiab] OR diagnose[tiab] OR diagnoses[tiab] OR diagnosed[tiab] OR diagnosing[tiab] OR diagnostic[tiab] OR diagnosis[tiab] OR audit[tiab] OR audits[tiab] OR auditing[tiab] OR audited[tiab] OR validate[tw] OR validates[tw] OR validated[tw] OR validating[tw] OR validation[tw] OR tool[tiab] OR tools[tiab] OR toolkit[tiab] OR toolkits[tiab] OR generalizable[tiab] OR generalizability[tiab]) AND ("tomography, x-ray computed"[MeSH Terms] OR ("tomography"[tiab] AND "x-ray"[tiab] AND "computed"[tiab]) OR "x-ray computed tomography"[tiab] OR ("computed"[tiab] AND "tomography"[tiab]) OR "computed tomography"[tw] OR "magnetic resonance imaging"[MeSH Terms] OR ("magnetic"[tiab] AND "resonance"[tiab]) OR "magnetic resonance imaging"[tiab] OR "mammography"[MeSH Terms] OR "mammography"[tiab] OR "mammographies"[tiab]) AND ("united states food and drug administration"[MeSH Terms] OR ("united"[All Fields] AND "states"[All Fields] AND "food"[All Fields] AND "drug"[All Fields] AND "administration"[All Fields]) OR "united states food and drug administration"[All Fields] OR "fda"[All Fields] OR "CE mark"[tw] OR "CE-mark"[tw] OR "commercial algorithm"[tw] OR "commercial product"[tw] OR "commercially available algorithm"[tw] OR "commercially available product"[tw] OR ("commercial"[All Fields] AND "availability"[All Fields]) OR ("commercially"[All Fields] AND "available"[All Fields] AND "product"[All Fields])) OR ("ADM Diagnostics"[tw] OR "Advantis"[tw] OR "AI Medical"[tw] OR "AI Metrics"[tw] OR "AI4MedImaging"[tw] OR "aidence"[tw] OR "Aidoc"[tw] OR "AIQ Solutions"[tw] OR "AIRAmed"[tw] OR "AlgoMedica"[tw] OR "AmCad BioMed"[tw] OR "annalise.ai"[tw] OR "Avenda Health"[tw] OR "Avicenna.AI"[tw] OR "Axial Medical"[tw] OR "AZmed"[tw] OR "b-rayZ"[tw] OR "Bay Labs"[tw] OR "Behold.ai"[tw] OR "BK Medical "[tw] OR "Body Vision Medical "[tw] OR "Brainminer"[tw] OR "Brainomix"[tw] OR "Brainreader"[tw] OR "BrainTale"[tw] OR "Bunkerhill"[tw] OR "Butterfly Network"[tw] OR "Caption Health"[tw] OR "Carebot"[tw] OR "Carestream Health"[tw] OR "Carina Medical"[tw] OR "CathWorks"[tw] OR "Ceevra"[tw] OR "Cercare Medical"[tw] OR "Cerebriu"[tw] OR "Circle Cardiovascular Imaging"[tw] OR "ClariPi"[tw] OR "Clarius Mobile Health"[tw] OR "ClearPoint Neuro"[tw] OR "ClearView Diagnostics"[tw] OR "Cleerly"[tw] OR "Combinostics"[tw] OR "contextflow"[tw] OR "Coreline Soft"[tw] OR "Cortechs"[tw] OR "CorticoMetrics"[tw] OR "CuraCloud"[tw] OR "Curemetrix"[tw] OR "Cycle Clarity"[tw] OR "Cydar "[tw] OR "Deep01 Limited"[tw] OR "DeepTrace Technologies"[tw] OR "DeepVoxel"[tw] OR "Delft Imaging"[tw] OR "Densitas"[tw] OR "DiA Imaging Analysis"[tw] OR "Digitec"[tw] OR "Dyad Medical"[tw] OR "EchoNous"[tw] OR "Eko Devices"[tw] OR "Elaitra"[tw] OR "Elekta Solutions"[tw] OR "Elucid Bioimaging"[tw] OR "Ever Fortune"[tw] OR "EXINI Diagnostics"[tw] OR "Ezra AI"[tw] OR "Feops"[tw] OR "Fluidda"[tw] OR "Formus Labs"[tw] OR "Galgo Medical"[tw] OR "Genesis Software Innovations"[tw] OR "GLEAMER"[tw] OR "Hanalytics"[tw] OR "HeartFlow"[tw] OR "HeartLung Corporation"[tw] OR "HeartVista"[tw] OR "Hera-MI"[tw] OR "HY Medical"[tw] OR "Hyperfine Research"[tw] OR "icometrix"[tw] OR "ImageBiopsy Lab"[tw] OR "Imagen Technologies"[tw] OR

"Imaging Biometrics"[tw] OR "Imbio"[tw] OR "IMedis"[tw] OR "Imeka Solutions"[tw] OR "Infervision"[tw] OR "Infinitt Healthcare"[tw] OR "JF Healthcare"[tw] OR "KeyaMed"[tw] OR "Kheiron Medical Technologies"[tw] OR "Koios Medical"[tw] OR "Ligence"[tw] OR "Limbus Ai"[tw] OR "Lucida Medical"[tw] OR "Lunit"[tw] OR "Manteia Technologies"[tw] OR "MaxQ-Al "[tw] OR "MedCognetics"[tw] OR "mediaire"[tw] OR "Medic Vision"[tw] OR "MEDICAL IP "[tw] OR "Medicalgorithmics"[tw] OR "Medimaps"[tw] OR "Medipixel"[tw] OR "Medis Medical Imaging"[tw] OR "Medviso"[tw] OR "Merge Healthcare"[tw] OR "MeVis"[tw] OR "Milvue"[tw] OR "MIM Software"[tw] OR "Mint Labs"[tw] OR "Mirada Medical "[tw] OR "LuCAS-plus"[tw] OR "Motilent"[tw] OR "MVision AI"[tw] OR "Nanox.AI"[tw] OR "NeoSoft"[tw] OR "Neosoma"[tw] OR "Neurophet"[tw] OR "Nicolab"[tw] OR "Olea Medical"[tw] OR "Omega Medical Imaging"[tw] OR "Optellum"[tw] OR "OrthoGrid Systems"[tw] OR "Oxipit"[tw] OR "OXOS Medical"[tw] OR "PAIRE"[tw] OR "Perspectum"[tw] OR "Quantitative Radiology Solutions"[tw] OR "Qubiotech"[tw] OR "Quibim"[tw] OR "Qure.ai"[tw] OR "QView Medical"[tw] OR "Qynapse"[tw] OR "Radformation"[tw] OR "Radiobotics"[tw] OR "RADLogics"[tw] OR "RapidAI"[tw] OR "Rayscape"[tw] OR "RaySearch Laboratories"[tw] OR "RebrAIn"[tw] OR "Resonance Health"[tw] OR "RevealDx"[tw] OR "Riverain Technologies"[tw] OR "ScanMed"[tw] OR "ScreenPoint Medical"[tw] OR "See-Mode Technologies"[tw] OR "Seno Medical Instruments"[tw] OR "SenseTime"[tw] OR "Shanghai United Imaging Healthcare"[tw] OR "Shenzhen Mindray Bio-Medical Electronics"[tw] OR "Smart Soft Healthcare"[tw] OR "SmarTarget"[tw] OR "Sonic incytes"[tw] OR "Sonio"[tw] OR "Spectrum Dynamics Medical"[tw] OR "Spimed-AI"[tw] OR "STATLIFE"[tw] OR "Surgical Information Sciences"[tw] OR "Synapsica"[tw] OR "SyntheticMR"[tw] OR "TaiHao Medical"[tw] OR "TeraRecon"[tw] OR "TheraPanacea"[tw] OR "Therapixel"[tw] OR "Thirona"[tw] OR "Tissue Differentiation Intelligence"[tw] OR "Tomtec Imaging Systems"[tw] OR "UltraSight "[tw] OR "Ultromics"[tw] OR "MX Healthcare"[tw] OR "Us2.ai"[tw] OR "Varian Medical Systems"[tw] OR "Ventripoint Diagnostics"[tw] OR "Verathon"[tw] OR "VinBrain"[tw] OR "Visage Imaging"[tw] OR "Visiana"[tw] OR "Visionairy Health"[tw] OR "Vital Images"[tw] OR "Viz.ai"[tw] OR "Volpara Health"[tw] OR "VUNO"[tw] OR "Vysioneer"[tw] OR "Whiterabbit.ai"[tw] OR "Xiamen Manteia Technology "[tw] OR "Yukun (Beijing) Technology Co"[tw] OR "Zepmed"[tw] OR "Ziosoft"[tw] OR "DeepHealth"[tw] OR "Quantib"[tw] OR "4Dmedical"[tw] OR "Matakina Technology "[tw] OR "iSchemaView"[tw])NOT (animals[MeSH Terms] NOT humans[MeSH Terms]) NOT (review[Publication Type]))

c) Code to extract DOI from Health Register without clicking every link:

```
const getPeerReviewed = () => {

  const rows = [...document.querySelectorAll('#evidence tbody tr')];
  console.log(rows.map(row => {
    const firstCell = row.querySelector('td');
    if( firstCell.innerText === "Peer reviewed papers on performance") {
      const paperSections = [...row.querySelectorAll('td')[1].querySelectorAll('ul li')];
      return paperSections.map( paperSection => {
        const sectionUrl = paperSection.querySelector('a').attributes.href.nodeValue;
        return `"${paperSection.innerText.replace('(read)', '')}",${sectionUrl}`;
      })
    }
```

```
        if( firstCell.innerText === "Non-peer reviewed papers on performance") {
            const paperSections = [...row.querySelectorAll('td')[1].querySelectorAll('ul li')];
            return paperSections.map( paperSection => {
                let sectionUrl = "[none]";
                const anchor = paperSection.querySelector('a');
                if(anchor.attributes.href != null) {
                    sectionUrl = anchor.attributes.href.nodeValue;
                }
                return `"${paperSection.innerText.replace('(read)', '')}",${sectionUrl}`;
            })
        }
        if( firstCell.innerText === "Other relevant papers") {
            const paperSections = [...row.querySelectorAll('td')[1].querySelectorAll('ul li')];
            return paperSections.map( paperSection => {
                let sectionUrl = "[none]";
                const anchor = paperSection.querySelector('a');
                if(anchor.attributes.href != null) {
                    sectionUrl = anchor.attributes.href.nodeValue;
                }
                return `"${paperSection.innerText.replace('(read)', '')}",${sectionUrl}`;
            })
        }

    }).filter(x => x != null).flatMap( p => p ).join('\n'));
}
getPeerReviewed();
```

d) Minimum sensitivity equation

Using the simple minimum formula $S_{min} = 1 - \frac{(1 - S_{overall})}{x}$ we can guess the minimum sensitivity for any subgroup that is x% of the population. The collected studies averaged 84% sensitivity with a median of 84% sensitivity.

Sex subgroup analysis:
If we apply this equation to the sex demographic, where male patients averaged 53% of the cohort:

$$S_{min} = 1 - \frac{(1 - 0.84)}{0.53}$$

S_{min} = 69.8%
Thus, the sensitivity for the male subgroup can be as low as 70% while maintaining an overall sensitivity of 84%.

If we apply this to the female patients, who averaged 47% of the cohort:

$$S_{min} = 1 - \frac{(1 - 0.84)}{0.47}$$

S_{min} = 65.96%
Thus, the sensitivity for the female subgroup can be as low as 66% while maintaining an overall sensitivity of 84%.

Race and ethnicity subgroup analysis:
If we instead apply the minimum estimation equation to the ethnoracial data collected, the minimum sensitivity quickly becomes clinically unacceptable, as shown in the table below.

		Overall sensitivity estimate			
	Normalized median % of cohort	80%	85%	90%	95%
% White or Caucasian	64.8%	69.1%	76.9%	84.6%	92.3%
% Asian	14.0%	0.0%	0.0%	28.7%	64.4%
% Black or African American	9.8%	0.0%	0.0%	0.0%	49.2%
% Hispanic	6.8%	0.0%	0.0%	0.0%	26.3%
% Other	4.5%	0.0%	0.0%	0.0%	0.0%

For example, White patients made up a normalized median of 60% of the datasets we collected but the next largest ethnic group, Asians, comprised a normalized median of 14.5% of all recorded datasets. Substituting 0.53 for 0.145 in the equation dramatically widens the sensitivity range for the Asian subgroup. This issue is further exaggerated with smaller subgroups.

e) Wilson CI equation

The number of patients per subgroup needed to attain a certain sensitivity can be roughly estimated using the Wilson confidence interval[1–3] equation:

$$n_{patients\ per\ balanced\ subgroup} = \frac{\left[z_{1-\alpha/2}\sqrt{2\underline{p}(1-\underline{p})} + z_{1-\beta}\sqrt{p_1(1-p_1)+p_2(1-p_2)}\right]^2}{(p_1-p_2)^2} \div prevalence$$

$z_{1-\alpha/2}$ = standard critical value for a 2-sided test where α is 0.05 = 1.96

$\underline{p}$ = target or overall sensitivity

$z_{1-\beta}$ = power quantile of 80% = 0.84

p_1 = target or overall sensitivity + the half width h

p_2 = target or overall sensitivity - the half width h

The Wilson CI formula works with any binomial value, so we can substitute sensitivity for accuracy or specificity if these are more appropriate for the model being validated. Adjusting for the slight imbalance

between the number of patients per sex didn't significantly affect the results, so we will use this simpler formula in the following example.

Following the work of Bosman, et al[4], if we assume 10% TB prevalence as they did, and a sensitivity near 90% as recommended by the WHO[5], with a precision of 5%, power at 80%, and a half-width of 5%, the Wilson CI formula gives us that each dataset must have at least 139 TB positive patients in both sex subgroups. Further, we know that there must be a total of 1399 patients per sex at this prevalence.

$$n_{patients\ per\ balanced\ subgroup} = \frac{\left[z_{1-\alpha/2}\sqrt{2(0.9)(1-0.9)} + z_{1-\beta}\sqrt{0.95(1-0.95)+0.85(1-0.85)}\right]^2}{(0.95-0.85)^2} \div prevalence$$

$$n_{patients\ per\ balanced\ subgroup} = \frac{\left[1.96\sqrt{2(0.9)(1-0.9)} + 0.84\sqrt{0.95(1-0.95)+0.1275)}\right]^2}{(0.95-0.85)^2} \div prevalence$$

$$n_{patients\ per\ balanced\ subgroup} = \frac{139.94}{prevalence}$$

$$n_{patients\ per\ balanced\ subgroup} = 1399$$

By this logic, we need about 278 total TB+ patients.

$$n_{male\ TB+\ patients} = 139$$

$$n_{female\ TB+\ patients} = 139$$

$$n_{male\ TB+\ patients} + n_{female\ TB+\ patients} = 278$$

And we need at least 2798 total patients.

$$n_{male\ patients} = 1399$$

$$n_{female\ patients} = 1399$$

$$n_{male\ patients} + n_{female\ patients} = 2798$$

The authors collected 1232 patients for this study, and 132 of these were determined to be TB positive.

$n_{patients\ collected} < n_{patients\ needed}$

$n_{TB+\ patients\ collected} < n_{TB+\ patients\ needed}$

Even without knowing how many of these positive cases were male or female patients, we know this study is likely underpowered for sex subgroup analysis since the total is fewer than the minimum required per subgroup.

For unbalanced groups like ethnoracial demographics, you can include the variable k, the number of patients in subgroup 2 divided by the number of patients in subgroup 1.

$$n_2 = k\,n_1 = \frac{\left[z_{1-\alpha/2}\sqrt{(1+1/k)*\underline{p}(1-\underline{p})} + z_{1-\beta}\sqrt{p_1(1-p_1)+\frac{p_2(1-p_2)}{k}}\right]^2}{(p_1-p_2)^2} \div prevalence$$

$$n_2 = k\,n_1 = \frac{\left[1.96\sqrt{(1+1/k)*\underline{p}(1-\underline{p})} + 0.84\sqrt{p_1(1-p_1)+\frac{p_2(1-p_2)}{k}}\right]^2}{(p_1-p_2)^2} \div prevalence$$

Supplementary Tables

Table S1: Studies that present both demographic details and subgroup analysis results for that demographic subgroup

DOI	Publication year	Subspecialty	Imaging Modality	Sponsorship by device maker	Authors have COI	No. of Patients	Includes Sex Ratios	Includes Age Ratios	Includes Race and ethnicity Ratios	Includes Sex-based performance data	Includes Age-based performance data	Include Race and ethnicity-based performance data
10.1016/j.acra.2010.06.007	2010	Skeletal	Radiography	unclear	1	708	1	0	1	1	0	1
10.1515/JPEM.2011.420	2011	Skeletal	Radiography	unclear	1	116	1	0	0	1	0	0
10.1097/rli.0b013e318262292e	2012	Lung	CT	0	0	92	1	1	1	1	1	N/A
10.1186/s13058-014-0439-1	2014	Breast	Mammography	0	0	1099	N/A	1	1	N/A	1	1
10.1093/eurheartj/ehv444	2015	Cardiology	CT	1	0	297	1	1	0	0	1	0
10.1002/jbmr.2734	2016	Skeletal	Radiography	0	1	17809	1	0	0	1	1	0
10.1016/j.jcct.2017.08.001	2017	Cardiology	CT	1	1	238	1	0	0	1	0	0
10.1007/s00414-016-1471-8	2017	Skeletal	Radiography	unclear	1	371	1	0	0	1	1	0
10.1093/eurheartj/ehy530	2018	Cardiology	CT	1	0	4737	1	0	0	1	1	0
10.1164/rccm.201704-0692OC	2018	Lung	CT	0	0	1184	1	0	1	1	1	0
10.3174/ajnr.A6303	2019	Brain	CT	0	1	58	1	0	0	1	1	0
10.1016/j.nicl.2019.101711	2019	Brain	MRI	0	1	200	1	1	0	0	1	0
10.1148/radiol.2019191225	2019	Lung	CXR	0	1	1135	1	1	0	1	1	0
10.1016/j.wneu.2020.05.121	2020	Brain	CT	unclear	0	26	1	1	0	1	1	0
10.1016/S2589-7500(20)30003-0	2020	Breast	Mammography	1	0	2362	N/A	1	0	N/A	1	0
10.1016/j.chest.2020.12.004	2020	Lung	CT	0	1	7262	1	0	1	1	1	1
10.1007/s00330-019-06589-8	2020	Lung	CXR	0	1	169	1	0	0	1	1	0
10.1016/S2589-7500(20)30221-1	2020	Lung	CXR	0	1	272	1	1	0	1	1	0
10.1097/MD.0000000000019870	2020	Lung	Ultrasound	0	0	57	1	0	0	1	0	0
10.1159/000518020	2021	Abdomen	MRI	0	0	8	1	1	0	1	1	0
10.1016/j.jacr.2021.03.005	2021	Brain	CT	0	0	3605	1	0	0	1	1	0
10.3389/fneur.2021.656112	2021	Brain	CT	unclear	1	814	1	1	0	1	1	0
10.1007/s00261-020-02595-3	2021	Liver	MRI	0	1	57	1	0	0	1	0	0
10.1007/s00330-020-07269-8	2021	Lung	CXR	0	0	697	1	0	0	1	1	0
10.1016/S2589-7500(21)00116-3	2021	Lung	CXR	0	0	23954	1	1	0	1	1	0
10.1038/s41598-021-03265-0	2021	Lung	CXR	0	0	1032	1	1	0	1	1	0
10.1016/j.diii.2021.10.007	2021	Skeletal	Radiography	0	0	2549	1	1	0	0	1	0
10.1016/j.ejrad.2022.110321	2022	Breast	Mammography	0	1	2396	N/A	1	0	N/A	1	0
10.1016/j.breast.2022.07.009	2022	Breast	Ultrasound	unclear	1	172	N/A	1	0	N/A	1	0
10.1155/2022/1368878	2022	Cardiology	MRI	0	0	142	1	0	0	1	1	0
10.1007/s00330-022-08645-2	2022	Lung	CT	0	0	4454	1	0	0	1	0	0
10.3390/diagnostics12102382	2022	Lung	CXR	0	1	2407	1	0	0	1	1	0

DOI	Publication year	Subspecialty	Imaging Modality	Sponsorship by device maker	Authors have COI	No. of Patients	Includes Sex Ratios	Includes Age Ratios	Includes Race and ethnicity Ratios	Includes Sex-based performance data	Includes Age-based performance data	Include Race and ethnicity-based performance data
10.1016/j.lana.2022.100388	2022	Lung	CXR	0	0	2075	1	0	1	N/A	1	1
10.1001/jamanetworkopen.2022.47172	2022	Lung	CXR	1	0	985	1	1	0	1	1	0
10.1016/j.ijid.2022.05.037	2022	Lung	CXR	0	0	2190	1	1	0	1	1	0
10.1093/cid/ciab639	2022	Lung	CXR	0	0	3727	1	1	0	1	1	0
10.1371/journal.pdig.0000067	2022	Lung	CXR	0	0	12890	1	1	0	1	1	0
10.3348/jksr.2021.0128	2022	Skeletal	Radiography	0	0	2126	1	1	0	1	0	0
10.1007/s00247-022-05496-3	2022	Skeletal	Radiography	0	1	300	1	1	0	0	1	0
10.1007/s00256-022-04070-0	2022	Skeletal	Radiography	1	1	300	1	1	0	0	1	0
10.1016/j.diii.2022.06.004	2022	Skeletal	Radiography	0	0	500	1	1	0	0	1	0
10.1016/j.dld.2022.10.015	2023	Abdomen	MRI	0	1	87	1	0	0	1	0	0
10.1136/jnis-2023-020445	2023	Brain	CT	0	1	360	1	0	0	1	1	1
10.1016/j.jacr.2023.04.003	2023	Brain	CT	0	0	1236	1	1	1	1	1	1
10.1016/j.ejrad.2023.111126	2023	Brain	MRI	0	1	995	1	1	0	1	1	0
10.1016/S2589-7500(23)00153-X	2023	Breast	Mammography	1	0	55581	N/A	1	0	N/A	1	0
10.2196/48123	2023	Breast	Mammography	unclear	1	10889	N/A	1	0	N/A	1	0
10.1200/JCO.22.01153	2023	Breast	Mammography	0	0	7407	N/A	1	1	N/A	1	1
10.1371/journal.pone.0287953	2023	Lung	CT	0	0	53	1	0	0	1	0	0
10.3390/diagnostics13071324	2023	Lung	CT	0	1	387	1	1	0	1	1	0
10.1016/j.rxeng.2022.11.007	2023	Lung	CXR	0	0	114	1	0	0	1	0	0
10.3348/kjr.2022.0651	2023	Lung	CXR	0	1	1761	1	0	0	1	1	0
10.3348/kjr.2023.0255	2023	Lung	CXR	0	1	3421	1	0	0	1	1	0
10.1148/radiol.221894	2023	Lung	CXR	0	1	5238	1	1	0	1	1	0
10.1371/journal.pgph.0000402	2023	Lung	CXR	0	0	2812	1	1	0	1	1	0
10.1002/jbmr.4916	2023	Skeletal	CT	0	1	1943	1	0	0	1	1	0
10.3390/life13010223	2023	Skeletal	Radiography	0	0	735	0	1	0	0	1	0
10.1007/s00330-023-10543-0	2023	Skeletal	Radiography	0	1	306	1	0	0	1	0	0
10.1016/j.diii.2023.04.003	2023	Skeletal	Radiography	1	0	206	1	0	0	1	0	0
10.3389/fendo.2023.1130580	2023	Skeletal	Radiography	0	1	1202	1	0	1	1	1	N/A
10.1016/j.acra.2023.06.016	2023	Skeletal	Radiography	0	1	1210	1	1	0	1	1	0
10.1016/j.wneu.2024.03.064	2024	Brain	CT	0	1	1789	0	1	0	0	1	0
10.1097/RLI.0000000000001122	2024	Brain	CT	0	0	3334	1	0	0	1	0	0
10.1148/radiol.232286	2024	Breast	Mammography	1	0	4855	N/A	0	1	N/A	1	1
10.1148/radiol.232303	2024	Breast	Mammography	0	1	2062	N/A	1	0	N/A	1	0
10.3390/diagnostics14171877	2024	Cardiology	CT	0	1	1303	1	0	0	1	1	0
10.1016/j.jcct.2024.01.016	2024	Cardiology	CT	0	1	102	1	1	0	1	1	0
10.3390/diagnostics14151635	2024	Cardiology	CXR	1	0	1117	1	0	0	1	0	0
10.1097/RTI.0000000000000746	2024	Lung	CXR	unclear	0	293	1	0	0	1	1	0

DOI	Publication year	Subspecialty	Imaging Modality	Sponsorship by device maker	Authors have COI	No. of Patients	Includes Sex Ratios	Includes Age Ratios	Includes Race and ethnicity Ratios	Includes Sex-based performance data	Includes Age-based performance data	Include Race and ethnicity-based performance data
10.3390/healthcare12070706	2024	Lung	CXR	0	0	477	1	0	0	1	1	0
10.1093/cid/ciae378	2024	Lung	CXR	0	1	1232	1	1	0	1	1	0
10.1016/j.ejrad.2024.111783	2024	Lung	CXR	unclear	0	20944	1	1	1	1	1	1
10.6065/apem.2346050.025	2024	Skeletal	Radiography	0	0	371	1	0	1	1	0	N/A
10.1016/j.acra.2023.10.042	2024	Skeletal	Radiography	1	0	2626	1	1	0	1	1	0
10.1093/bjro/tzae011	2024	Skeletal	Radiography	1	1	334	1	1	0	1	1	0
10.2214/AJR.24.31067	2024	Various	CT	0	unclear	2886	1	0	0	1	0	0
10.1148/radiol.230981	2024	Various	Radiography	unclear	1	712	1	1	0	1	1	0

References

1. Wilson EB (1927) Probable inference, the law of succession, and statistical inference. J Am Stat Assoc 22:209–212

2. Agresti A, Coull BA (1998) Approximate is better than “exact” for interval estimation of binomial proportions. Am Stat 52:119–126

3. Newcombe RG (1998) Interval estimation for the difference between independent proportions: comparison of eleven methods. Stat Med 17:873–890

4. Bosman S, Ayakaka I, Muhairwe J, et al (2024) Evaluation of C-reactive protein and computer-aided analysis of chest X-rays as tuberculosis triage tests at health facilities in Lesotho and South Africa. Clin Infect Dis 79:1293–1302

5. (2021) World Health Organization. WHO consolidated guidelines on tuberculosis. World Health Organization, Geneva, Switzerland
